# Anthrax Lethal Toxin Impairs IL-8 Expression in Epithelial Cells through Inhibition of Histone H3 Modification

**DOI:** 10.1371/journal.ppat.1000359

**Published:** 2009-04-03

**Authors:** Benoit Raymond, Eric Batsche, Florence Boutillon, Yong-Zheng Wu, Dominique Leduc, Viviane Balloy, Eloïse Raoust, Christian Muchardt, Pierre L. Goossens, Lhousseine Touqui

**Affiliations:** 1 Institut Pasteur, Unité de Défense Innée et Inflammation, Paris, France; 2 Unité Inserm, U.874, Paris, France; 3 Institut Pasteur, Unité de Régulation Epigénétique, Paris, France; 4 Institut Pasteur, Unité des Toxines et Pathogénie Bactérienne, Paris, France; 5 CNRS, URA-2172, Paris, France; University of Illinois, United States of America

## Abstract

Lethal toxin (LT) is a critical virulence factor of *Bacillus anthracis*, the etiological agent of anthrax, whose pulmonary form is fatal in the absence of treatment. Inflammatory response is a key process of host defense against invading pathogens. We report here that intranasal instillation of a *B. anthracis* strain bearing inactive LT stimulates cytokine production and polymorphonuclear (PMN) neutrophils recruitment in lungs. These responses are repressed by a prior instillation of an LT preparation. In contrast, instillation of a *B. anthracis* strain expressing active LT represses lung inflammation. The inhibitory effects of LT on cytokine production are also observed *in vitro* using mouse and human pulmonary epithelial cells. These effects are associated with an alteration of ERK and p38-MAPK phosphorylation, but not JNK phosphorylation. We demonstrate that although NF-κB is essential for IL-8 expression, LT downregulates this expression without interfering with NF-κB activation in epithelial cells. Histone modifications are known to induce chromatin remodelling, thereby enhancing NF-κB binding on promoters of a subset of genes involved in immune response. We show that LT selectively prevents histone H3 phosphorylation at Ser 10 and recruitment of the p65 subunit of NF-κB at the IL-8 and KC promoters. Our results suggest that *B. anthracis* represses the immune response, in part by altering chromatin accessibility of IL-8 promoter to NF-κB in epithelial cells. This epigenetic reprogramming, in addition to previously reported effects of LT, may represent an efficient strategy used by *B. anthracis* for invading the host.

## Author Summary

*Bacillus anthracis*, the etiological agent of anthrax, can infect mammals either accidentally or as a potential consequence of a terrorism threat. Pulmonary infection is a life-threatening form of the disease, causing a near 100% mortality rate in the absence of appropriate therapy. Thus, it is important to understand the mechanisms of host defense against *B. anthracis*. We examined the effects of various *B. anthracis* strains on lung inflammation in a mouse model of pulmonary anthrax and on human lung epithelial cells, the first barrier of lung against invading pathogens. We showed that a *B. anthracis* strain expressing lethal toxin inhibits inflammation. In contrast, a strain in which this toxin has been inactivated induces lung inflammation. We next examined the mechanisms involved in the inhibitory effect of lethal toxin. We showed that *B. anthracis* injects lethal toxin into epithelial cells, blocks the molecules associated on the chromosome, and thus represses production of mediators involved in inflammation. As the latter is a key process in host defense, its alteration by lethal toxin predisposes the host to infection by *B. anthracis*. This effect on the chromosomal machinery may represent an efficient strategy used by *B. anthracis* for invading the host.

## Introduction

Pulmonary infection by *B. anthracis*, the etiologic agent of anthrax disease [1], has been shown to be the most life-threatening form of the disease as compared to gastrointestinal and cutaneous forms of anthrax. Whatever the infection route used by this bacterium, spores are taken up by macrophages and/or dendritic cells, and subsequently migrate in the draining lymph nodes where they germinate [2],[3]. Germination leads to bacterial multiplication and dissemination through the whole organism. Despite a therapeutic intervention, all forms of infection may progress to fatal systemic anthrax, which is characterized by shock-like symptoms, sepsis, and respiratory failure [4]. Therefore, investigation of the immune response triggered by *B. anthracis* may help to better understand the pathophysiology of anthrax and to develop efficient therapeutic approaches for the treatment of this disease.

The innate immune response, including the inflammatory reaction that is the first line of host defense against invading pathogens, is characterized by upregulation of various inflammatory genes due to the activation of transcription factors. IL-8 (Interleukine-8, CXCL8) is a human chemokine that induces the recruitment of polymorphonuclear (PMN) neutrophils from the blood to the injured tissue [5]. This allows PMN to eradicate the invading pathogen within the site of infection. In healthy tissues, IL-8 is barely detectable, but is rapidly induced by nuclear factor κB (NF-κB) and activator protein 1 (AP-1)-dependent inflammatory stimuli such as TNFα, bacteria or virus [6]. Recently, significant advances in the understanding of signaling pathways, which regulate IL-8 transcription as well as mRNA stabilization in response to external stimuli, have been made [6]. It is now clearly established that induction of inflammatory genes is associated with local changes in histone modification that plays a major role in epigenetic regulation of gene expression. The core histones including H2A, H2B, H3 and H4 are the basic components of the nucleosomes that organize the cellular DNA in chromatin [7],[8]. Covalent modifications of the histones play a major role in gene regulation by affecting chromatin compaction and thereby DNA accessibility. These modifications can result in either transcriptional repression (like for H3me3K9 and H3me1K27) or activation (H3me3K4, H3pS10 and acetylation at various positions) [9],[10].

The exact mechanisms by which *B. anthracis* induces anthrax disease are not fully understood; however, it is clearly established that this bacterium spreads rapidly into the host at the early stages of infection with low or no detectable immune response [2],[11],[12]. This is likely due to the ability of *B. anthracis* to subvert the host immune response [11]–[13] *via*, at least in part, the action of tow major toxins, edema toxin and lethal toxin (LT). *B. anthracis* secretes a transporter called protective antigen (PA) which acts in pair with lethal factor (LF) leading to the formation of lethal toxin (LT). LT cleaves MAPKK thereby interfering with MAPK cascade [14],[15] a process known to play a major role in pathogenesis of anthrax. However, the molecular mechanisms that may link MAPK inhibition by LT and modulation of target gene expression remained unclear.

We report here that LT impaired the inflammatory response in a mouse model of pulmonary anthrax. Remarkably, LT repressed IL-8 expression without interfering with NF-κB activation, an essential transcription factor for IL-8 expression. Investigation of the molecular mechanisms involved in this inhibition revealed that LT blocked histone (H3) phosphorylation at serine 10, a modification normally induced by activation of the MAPK pathway and that is also involved in chromatin accessibility for NF-κB. This epigenetic reprogramming is probably a key mechanism by which *B. anthracis* circumvents the immune response during the earlier stage of infection.

## Results

### LT impairs *B. anthracis*–induced lung inflammatory response

**Figure 1:**
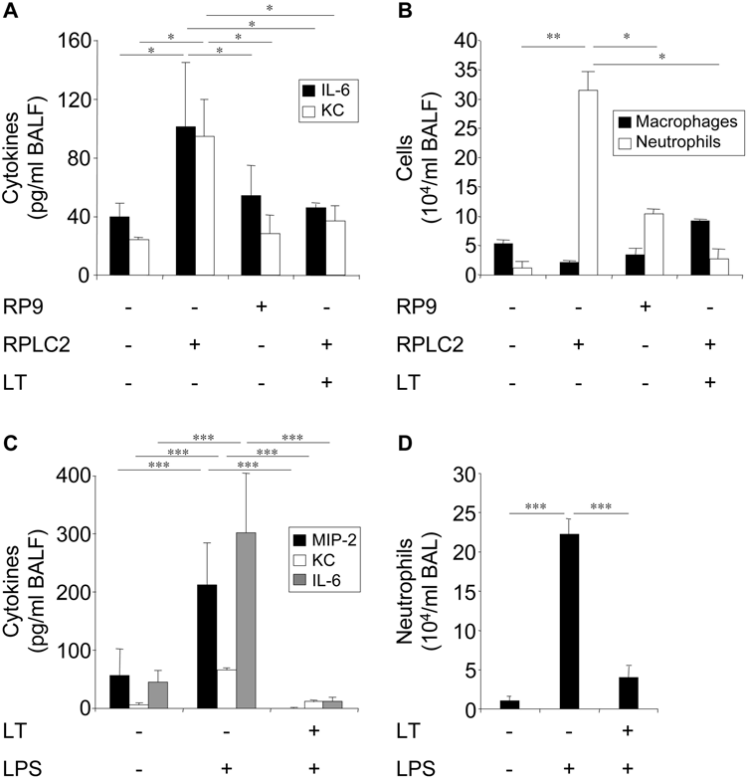
LT impairs pulmonary inflammatory response. (A,B) Mice received intratracheal instillation of LT (550 µg/kg) or saline 1 h before intranasal instillation of germinated RP9 or RPLC2 bacilli (10^7^ cfu). Sixteen hours later, BALs were performed, then KC and IL-6 concentrations (A), and the numbers of PMN neutrophils (B) were measured. (C,D) Mice received intratracheal instillation of LT (550 µg/kg) or saline 1 h before intranasal instillation of LPS (330 µg/kg). Sixteen hours later, BALs were performed, and the concentrations of MIP-2, KC, and IL-6 (C) and numbers of PMN neutrophils (D) were determined.

We examined the effect of *B. anthracis* on lung inflammation in a mouse model of pulmonary anthrax and the contribution of LT to this effect. All strains used in this study were in bacilli form. The degree of inflammatory reaction was assessed by measuring the recruitment of polymorphonuclear (PMN) leukocyte, and the levels of proinflammatory cytokines interleukin-6 (IL-6) and KC (mouse functional orthologue of human IL-8) in broncho-alveolar lavage fluids (BALFs). Intranasal instillation of the RPLC2 strain (producing inactive LT) induced an increase of IL-6 and KC levels (Figure 1A) and PMN recruitment (Figure 1B), in contrast to the RP9 strain (producing active LT) which had no effect. Intratracheal instillation of LT to mice before bacterial administration impaired RPLC2-induced increases of IL-6 and KC levels (Figure 1A) and PMN recruitment (Figure 1B). Intranasal instillation of LPS (instead of bacteria) induced an increase in concentrations of MIP-2, KC and IL-6 levels (Figure 1C) and PMN recruitment (Figure 1D). All these responses were abrogated by intratracheal instillation of LT before LPS.

### LT modulates cytokine expression by lung epithelial cells

**Figure 2:**
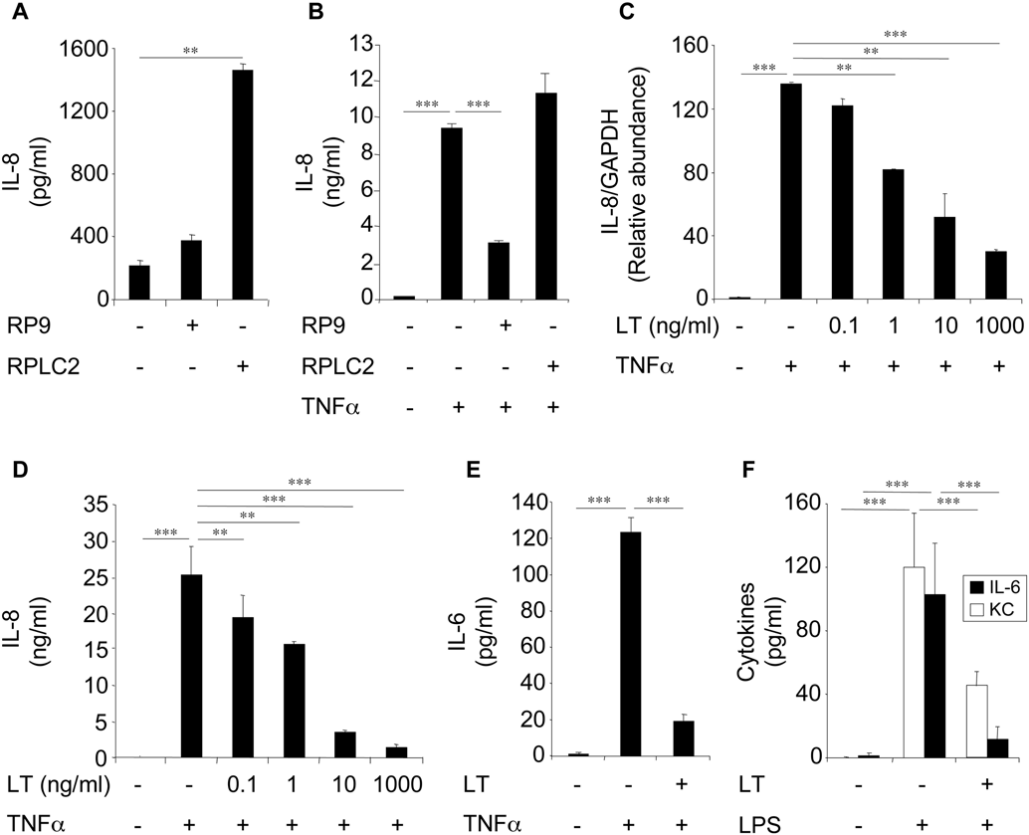
LT modulates cytokine expression by epithelial cells. (A,B) Beas-2B cells were infected with the *B. anthracis* strains RP9 and RPLC2 at an MOI of 20∶1 for 3 h in the absence of antibiotics. Cells were then washed and incubated in a medium supplemented with antibiotics in the absence (A) or presence (B) of TNFα. After 24 h incubation, supernatants were collected and IL-8 concentrations were measured. (C,D) Beas-2B cells were pretreated with LT (PA+LF) for 1 h before stimulation with TNFα (10 ng/ml). A fixed concentration of PA (1 µg/ml) and increasing concentrations of LF were used. After 24 h incubation, IL-8 mRNA expression (C) and IL-8 secretion (D) were measured. (E) Beas-2B cells were pretreated with LT (1 µg/ml) for 1 h before stimulation with TNFα (10 ng/ml), and IL-6 concentrations were measured after 24 h incubation. (F) Epithelial cells were isolated from lungs of naive mice and pretreated with LT (1 µg/ml) for 1 h before incubation with LPS (1 µg/ml). KC and IL-6 secretions were measured after 24 h stimulation.

These findings led us to examine the effect of LT on the *in vitro* secretion of cytokines by primary cells isolated from mouse lungs. LT blocked LPS-induced KC and IL-6 secretions by primary epithelial cells (Figure 2F) and MIP-2, KC and IL-6 secretions by alveolar macrophage (AMs) (data not shown).

We next investigated in more details the effects of LT on cytokine production in a human epithelial cell line Beas-2B. The RPLC2 strain induced IL-8 secretion by Beas-2B cells in contrast to the RP9 strain (Figure 2A). The RP9 strain inhibited TNFα-induced IL-8 secretion, whereas the RPLC2 strain had no effect (Figure 2B). LT blocked TNFα-induced IL-8 mRNA expression (Figure 2C), IL-8 (Figure 2D) and IL-6 (Figure 2E) secretions. Inhibition of IL-8 secretion by LT was also observed when Beas-2B cells were stimulated by IL-1β or LPS instead of TNFα (data not shown).

### Inhibition of IL-8 expression by LT is not due to an alteration of NF-κB activation/translocation

**Figure 3:**
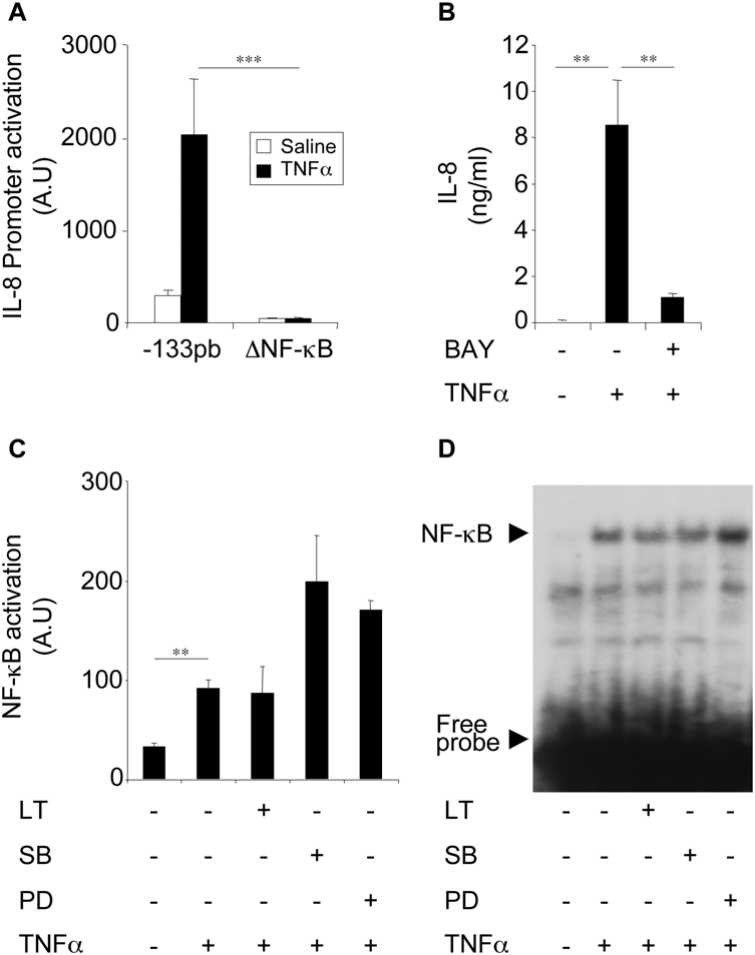
LT inhibits IL-8 expression through a process independent of NF-κB activation. (A) Beas-2B cells were transfected with IL-8 promoter (−133 pb) or [ΔNF-κB] IL-8 promoter reporter plasmids before stimulation with TNFα (10 ng/ml). Luciferase activity was measured after 24 h stimulation. (B) Cells were pretreated with BAY (10 µM) for 1 h and stimulated with TNFα (10 ng/ml). IL-8 concentrations were measured in supernatants after 24 h stimulation. (C) Cells were pretreated for 1 h with LT (1 µg/ml), SB203580, or PD98059 and incubated with TNFα (10 ng/ml). NF-κB luciferase activity was analysed after 24 h stimulation. (D) Cells were pretreated with LT (1 µg/ml), SB203580 (10 µM), or PD98059 (10 µM) and then stimulated with TNFα (10 ng/ml) for an additional 2 h. NF-κB translocation was analysed by EMSA.

As NF-κB is a key transcription factor of inflammatory gene expression including IL-8 [8], we examined its implication in IL-8 expression in our cell system. We analyzed IL-8-promoter activation in Beas-2B cells transfected with the (−133 pb) IL-8-promoter or an [ΔNF-κB] IL-8 promoter in which the responsive elements of NF-κB were mutated. TNFα induced the activation of the (−133 pb) IL-8-promoter but failed to stimulate the activation of the [ΔNF-κB] IL-8 promoter (Figure 3A). Preincubation of cells with BAY, an NF-κB inhibitor, abrogated TNFα-induced IL-8 secretion (Figure 3B). These findings indicated that NF-κB is essential for IL-8 expression in Beas-2B cells. The use of the [ΔAP-1] and [ΔNF-IL6] IL-8 promoter showed that AP-1 and NF-IL6 are only marginally involved in the induction of the IL-8 expression by TNFα (data not shown).

These findings led us to examine whether inhibition of IL-8 expression by LT is due to an alteration of NF-κB activation. Our results showed that neither LT nor MAPK inhibitors inhibited TNFα induced NF-κB activation or translocation. MAPK inhibitors even enhanced this activation (Figure 3C and 3D).

### LT inhibits IL-8 expression *via* a MAPK-dependent pathway

**Figure 4:**
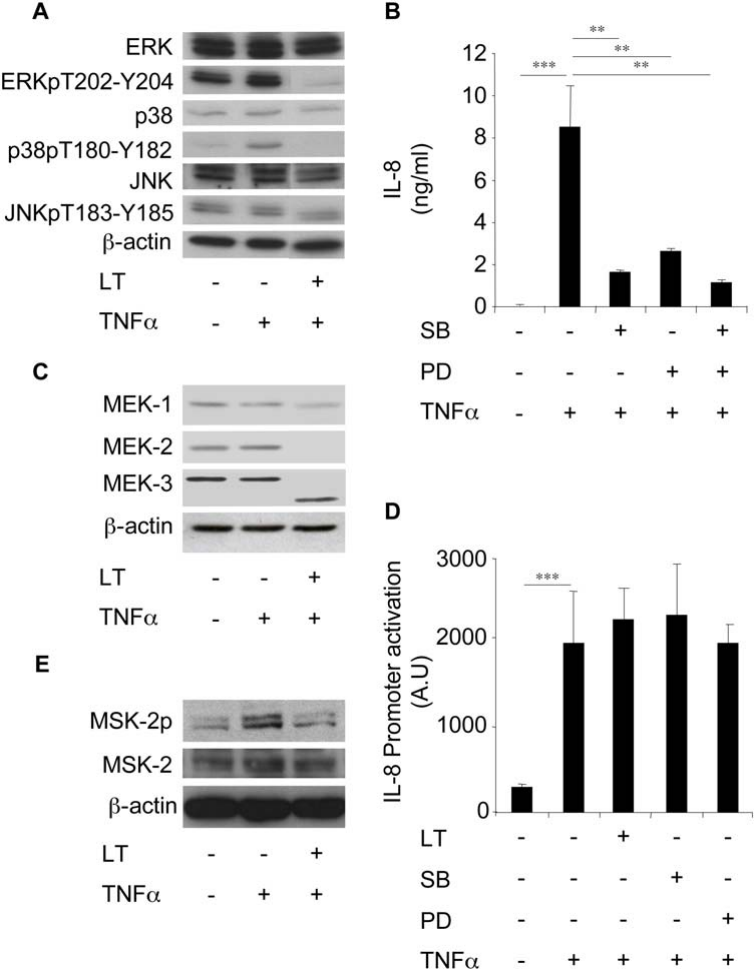
LT inhibits IL-8 expression *via* a MAPK-dependent pathway in Beas-2B cells. (A) Beas-2B cells were incubated for 1 h with LT (1 µg/ml) and stimulated with TNFα (10 ng/ml) for an additional 2 h. Western blot analyses were performed using antibodies directed against ERK, phospho-ERK (ERKpT202-Y204), p38, phospho-p38 (p38pT180-Y182), JNK, and phospho-JNK (JNKpT183-Y185) and were normalized with ß-actin antibody. (B) Cells were pretreated with SB203580 (10 µM) or PD98059 (10 µM) for 1 h before incubation with TNFα (10 ng/ml). IL-8 concentrations were measured in supernatants after 24 h stimulation. (C) Cells were incubated for 1 h with LT (1 µg/ml) and stimulated by TNFα (10 ng/ml) for an additional 2 h. Western blot analyses were performed using antibodies directed against MEK-1, MEK-2, and MEK-3 and were normalized with ß-actin antibody. (D) Cells were transfected with an IL-8 promoter reporter plasmid, and then incubated for 1 h with LT (1 µg/ml), SB203580 (10 µM), or PD98059 (10 µM) before addition of TNFα (10 ng/ml). Luciferase activity was then measured after 24 h stimulation. (E) Cells were incubated for 1 h with LT (1 µg/ml) and stimulated by TNFα (10 ng/ml) for an additional 45 min. Western blot analyses were performed using antibodies directed against MSK-2 and phospho-MSK-2 and normalized with ß-actin antibody.

MAPK cascade is known to modulate IL-8 expression in various cell systems [8]. This was confirmed in the present study using SB202190 and PD98059, specific inhibitors of p38-MAPK and ERK, respectively. Indeed, pre-treatment of Beas-2B cells with these compounds, significantly reduced TNFα-induced IL-8 expression (Figure 4B). We also showed that LT cleaves MEK1, MEK2, MEK3 in these cells (Figure 4C), a finding in agreement with the know ability of LT to cleave MAPKKs [14],[15]. LT inhibited TNFα-induced ERK and p38-MAPK phosphorylation, but had no effect on JNK phosphorylation (Figure 4A). Studies on Beas-2B cells transfected with the IL-8 promoter reporter plasmid showed that LT and MAPK inhibitors had no effect on TNFα-induced IL-8 promoter activity (Figure 4D), which contrasted with the ability of these compounds to inhibit the expression of endogenous IL-8. We next examined the effect of LT on the activation of mitogen and stress-activated kinases (MSK), located down-stream MAPKK pathways, and involved in histone phosphorylation. Incubation of Beas-2B cells with TNFα increased the level of MSK2 phosphorylation which was reduced by pre-treating cells with LT (Figure 4E). No detectable expression of MSK1 or phospho-MSK1 was observed in these cells (data not shown).

### LT impairs histone H3S10 phosphorylation and IL-8 and KC promoter accessibility

**Figure 5:**
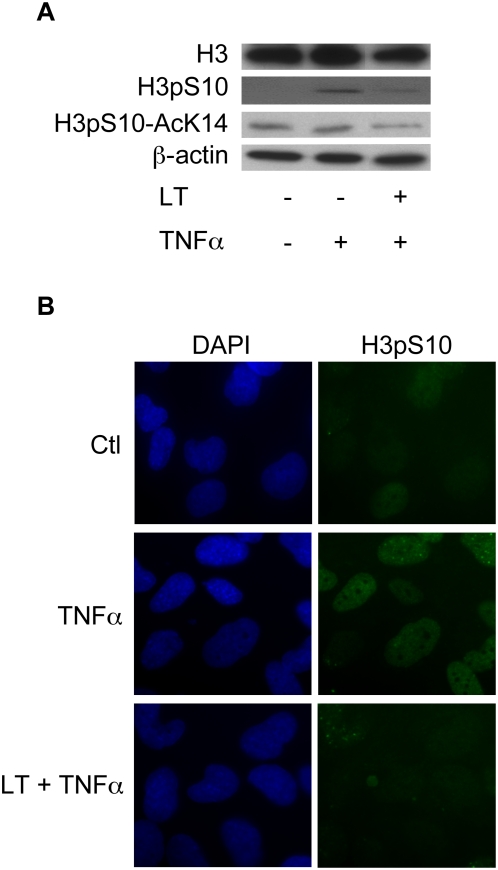
LT impairs H3 phosphorylation. Beas-2B cells were incubated for 1 h with LT (1 µg/ml) and stimulated with TNFα (10 ng/ml) for an additional 2 h. Western blot analyses were performed using H3, H3pS10, and H3pS10-AcK14 antibodies and were normalized with a β-actin antibody (A). Immunofluorescence analyses were performed using an H3pS10 antibody and DAPI (B).

**Figure 6:**
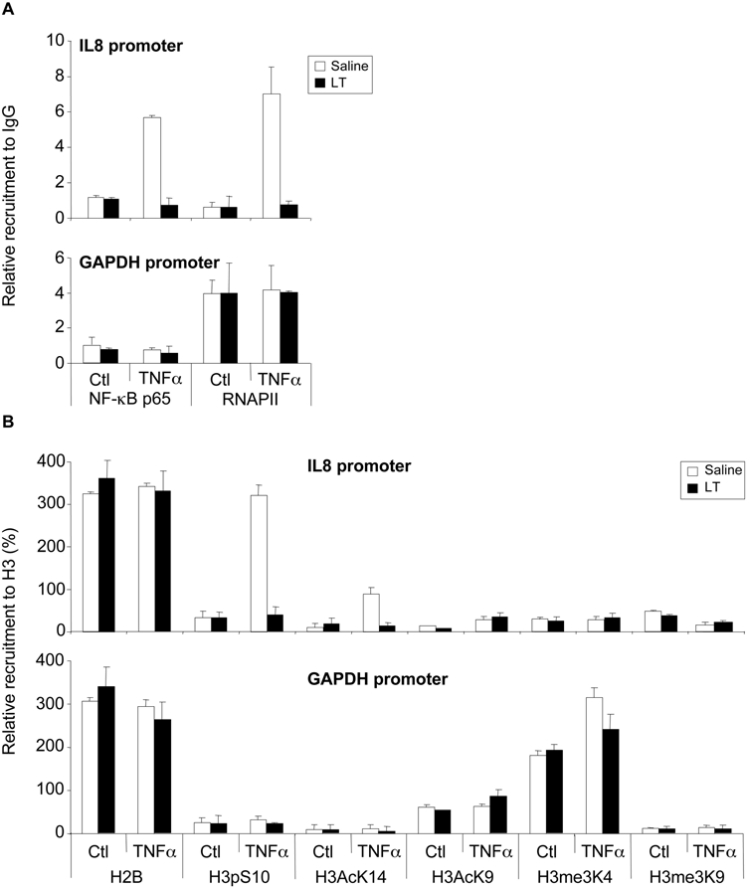
LT impairs IL-8 promoter accessibility. Beas-2B cells were incubated for 1 h with LT (1 µg/ml) and stimulated with TNFα (10 ng/ml) for additional 45 min. Cells were fixed with formaldehyde and then chromatin sonicated. The relative recruitment of NF-κB-p65 (p65) and the RNAPII (RNA Polymerase II) ChIP were expressed relative to the non-immun IgG rabbit used as a negative control (A). The H2B and the H3 histone tail modifications ChIP were reported to the H3 ChIP and expressed in percentages (B). Each value was corrected for the recruitment of each antibody on the CD44 exon 2 region used as an unregulated control gene by the TNFα and the LT. Note that the H2B signal relative to H3 is constant upon treatment and the promoters. It was three times more important than H3, probably because of the relative efficiency of antibodies.

The results above suggested that LT inhibited IL-8 expression, at a site probably located down-stream of NF-κB activation/translocation and depending on MAPK cascade. This led us to examine the effect of LT on the phosphorylation of H3, known to promote the accessibility of NF-κB to target promoters [5]–[8]. Western blot and immunofluorescence analyses showed that LT suppressed TNFα-induced H3 phosphorylation at Ser10 and acetylation at Lys14 (Figure 5A and 5B) in Beas-2B cells, suggesting that LT can impair the chromatin accessibility of NF-κB at the IL-8 promoter. We stimulated Beas-2B by TNFα with or without LT and then carried out chromatin immunoprecipitation (ChIP) assays using an antibody against p65 subunit of NF-κB, RNAPII antibodies or using an irrelevant immunoglobulin G (IgG) of the same isotype as a control. ChIP analyses showed that LT suppressed TNFα-induced p65 and RNAPII recruitment to the IL-8 promoter while no effect was observed in the recruitment at the GAPDH promoter (Figure 6A). In addition, the results showed that TNFα induced phosphorylation at Ser10 and acetylation at Lys14 of histone H3 on the IL-8 promoter whereas the other modifications on H3 were not significantly regulated. LT abrogated TNFα-induced presence of di-modified histone H3 phosphorylated at Ser10 and acetylated at Lys14 at the IL-8 promoter (Figure 6B). Acetylation at Lys9 and tri-methylation at Lys4 (known to reflect positive modulation of transcription) were not modified on the IL-8 promoter in our experimental conditions. Tri-methylation at Lys9, which generally correlates with inhibition of transcription, occurred in parallel to tri-methylation at Lys4 (Figure 6B).

**Figure 7:**
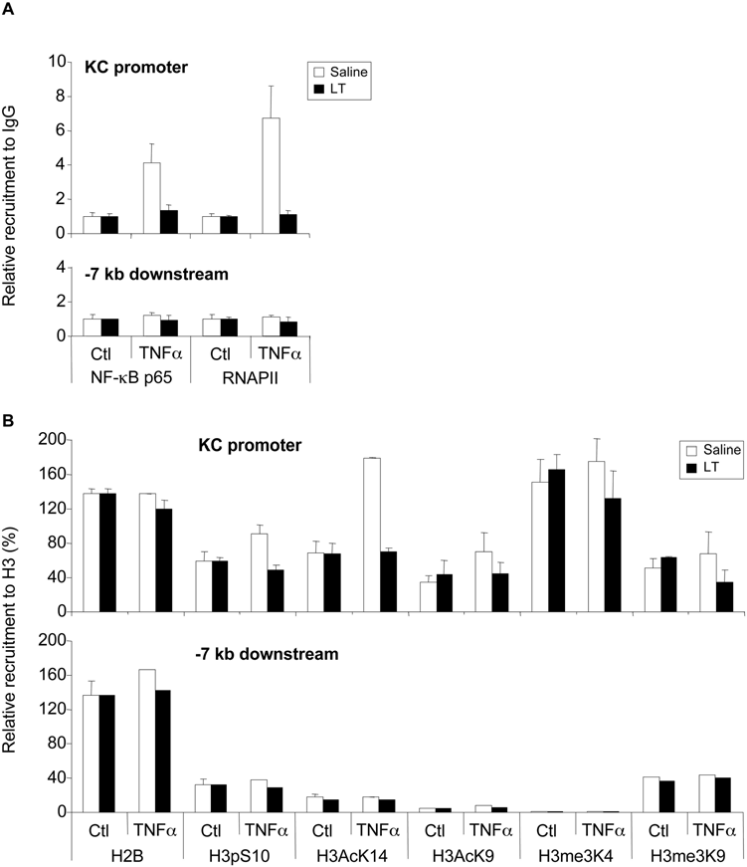
LT impairs KC promoter accessibility. MLE-15 cells were incubated for 1 h with LT (1 µg/ml) and stimulated with TNFα (10 ng/ml) for an additional 45 min. Cells were fixed with formaldehyde and then chromatin sonicated. The relative recruitment of NF-κB-p65 (p65) and the RNAPII (RNA Polymerase II) ChIP were expressed relative to the non-immun IgG rabbit used as a negative control (A). The H2B and the H3 histone tail modifications ChIP were reported to the H3 ChIP and expressed in percentages (B). Each value was corrected for the recruitment of each antibody on the CD44 exon 2 region used as an unregulated control gene by TNFα and LT. Note that the H2B signal relative to H3 is constant upon treatment and the promoters. It was two times more important than H3, probably because of the relative efficiency of antibodies.

We next performed ChIP analyses on a mouse epithelial cell line MLE-15 to examine the effect of LT on the KC promoter. We verified that LT inhibited TNFα-induced KC expression in these cells (data not shown). We observed almost similar regulations of histone modifications in this promoter (Figure 7). The weaker effects on KC versus IL-8 of the TNFα treatment on the H3pS10 and AcK14 modifications could be explained partially by the fact that the KC promoter appears to contain more H3me3K4 than the IL-8 promoter (160% of H3 versus 40% of H3, respectively), suggesting that the KC promoter chromatin could be in a more opened configuration than the IL-8 one.

## Discussion

Pulmonary anthrax is an acute disease of animals and humans caused by inhalation of spores of pathogenic strains of *B. anthracis* [1]–[3]. Upon infection, the spore germinates and begins to outgrow into toxin-producing replicating bacilli, which can ultimately kill the infected host [2],[11],[12]. LT, a major virulence factor of *B. anthracis*, has been shown to play a key role in the pathophysiology of anthrax and associated mortality [11],[12]. We demonstrated here that *B. anthracis* represses the mouse pulmonary inflammatory response (PMN recruitment and cytokine production) and that LT plays a key role in this inhibition. Moreover, in the absence of LT, *B. anthracis* induces this response, which suggests that component(s) of this bacterium is (are) able to induce an inflammatory reaction, and that their action is masked by the inhibitory effect of LT. This is consistent with previous studies, which have reported that *B. anthracis* spores stimulate cytokine production in various cells [16]–[18]. Our recent study showed that peptidoglycan, a constitutive cell wall component of *B. anthracis*, induces type-IIA secreted phospholipase A2 expression in AMs through a NF-κB dependent process [13]. In the earlier stages of infection, inhaled spores trigger an inflammatory response that is subsequently repressed as soon as LT is produced by outgrowing bacilli. Therefore, it was of a great interest to identify the molecular mechanisms by which LT represses the expression of genes involved in the inflammatory response.

Early studies demonstrated that phosphorylation of histones at specific amino acid residues regulates gene transcription [7]–[10]. The present study suggested that *B. anthracis* targets chromatin access for transcription factors, which may alter gene transcription. LT has been shown to suppress cytokine production by a process involving the disruption of MAPKKs [11],[12], yet the molecular mechanisms by which this disruption inhibits the expression of inflammatory genes remained to be elucidated. We reported here for the first time that inhibition of cytokine production in epithelial cells by LT occurs *via* a process involving an alteration of histone H3 phosphorylation/acetylation. Histone modification such as phosphorylation of H3 and acetylation of lysine on histones H3 or H4 can be associated with transcriptional activation [19]–[22]. Indeed, gene expression occurs when the chromatin is in the opened conformation that is promoted in part by histone phosphorylation. Moreover, phosphorylation of H3 is mediated, in part, by mitogen and stress-activated kinase (MSK), which is activated by MAPK (p38-MAPK and ERK) signaling pathways [22],[23]. Our findings showed that LT blocked the activation of p38-MAPK and ERK (as a consequence of MAPKK cleavage by LT) and that this inhibition led to the alteration of histone H3 phosphorylation through a process involving an inhibition of MSK2 phosphorylation. Indeed, MSK2 has been shown to mediate histone H3 phophorylation [22],[23].

The present study shows that both MAPK and NF-κB pathways are essential for IL-8 expression in Beas-2B cells. These results are consistent with previous reports showing that these pathways play a major role in the induction of IL-8 expression in various cell systems [6]. Our results showed that NF-κB activation in Beas-2B cells occurred *via* a MAPK-independent process. This led us to suggest that MAPK act down-stream of NF-κB activation to modulate IL-8 expression. Our results suggested that epigenetic remodeling initiated by MAPK-induced H3 phosphorylation promotes the accessibility of IL-8 promoters to NF-κB, thereby enhancing IL-8 expression. This process seems also to play a role in the modulation of KC promoter activity, the mouse functional orthologue of human IL-8 promoter. This epigenetic modulation, which is in part under the control of MAPKs, may occur only in the chromatin context whose remodeling favors the recruitment of transcription machinery components including NF-κB. This may explain why MAPK inhibition failed to interfere with the activation of the transfected IL-8 promoter reporter construct, which is not in the context of chromatin and therefore cannot be modulated *via* a MAPK-dependent epigenetic remodeling. Our results are in agreement with the recent study of Arbibe *et al* [24] showing that OspF, a virulence factor of *Shigella flexneri* endowed with phosphatase activity, reduced H3 phosphorylation on a limited subset of gene promoters, including IL-8 promoter, which then become inaccessible to NF-κB. Although both *S. flexneri* and *B. anthracis* downregulate inflammatory gene expression, they clearly interfere with H3 phosphorylation at different levels: *S. flexneri* acts downstream H3 phosphorylation by dephosphorylating ERK p38-MAPKs once they translocate into nucleus whereas *B. anthracis* blocks this process upstream these MAPKs by a cleavage of MAPKKs in the cytosol. However, it should be noted that, in addition to its epigenetic effect on the transcriptional activity of the IL-8 promoter, LT can also inhibit IL-8 expression via destabilization of IL-8 mRNA [25].

Recent studies have shown that lung epithelial cells interact with *B. anthracis* suggesting that these cells may play a role in host defense against pulmonary anthrax. Indeed, *B. anthracis* spores are taken up by lung epithelial cells either *in vitro* [26] and *in vivo* [27] and this uptake is followed by entry and germination of spores, though with low efficiency [26]. AMs and dendritic cells are also known to participate in host defense against inhaled *B. anthracis*, and play a role in spore uptake and germination [2],[3]. Interestingly, recent reports showed that spore germination and bacterial growth occurred at the site of entry, i.e. nasopharynx and alveoli [28]–[31]. Thus, available data suggest that spore germination, initial bacterial multiplication and lung epithelium crossing may occur along the respiratory tract. The respective role of each cell population still remains to be evaluated *in vivo*, in particular whether efficiency in spore uptake is paralleled with efficient dissemination from the lung. On the other hand, inhibition by toxins of the inflammatory response initiated by lung epithelial cells may favor the implication of AMs and dendritic cells in subsequent dissemination at later stages of the disease. Moreover, induction of cytokine production by epithelial cells has been shown to occur through a MAPK-dependent pathway [32]. Another report [33] showed that bronchial epithelial cells highly expressed TEM8, a receptor of *B. anthracis* toxins, thus confirming the implication of these cells in host response to *B. anthracis*. Lung epithelial cells have been shown to contribute to the innate immune response after interaction with pathogens such as virus [34] and bacteria [35]. Given the role of lung epithelial cells as an important source of chemokines (including IL-8), the inhibition of their activation by LT may have a critical impact on PMN recruitment, and therefore would compromise host defense against inhaled *B. anthtacis*. Indeed, a previous study showed that PMN play a role in host defense in pulmonary anthrax since PMN-depleted mice exhibited higher mortality by inhalation of *B. anthracis* [36]. Although lung epithelial cells could play a role during the activation of immune response in the early steps of anthrax infection, it should be, however, kept in mind that other pulmonary cell types such AMs, whose cytokine production is repressed by LT (our unpublished data), may contribute to the inhibitory effect of LT on lung IL-8/KC production.

In conclusion, we report here that *B. anthracis* represses the expression of cytokines such as IL-8 in epithelial cells. This inhibition occurs through a process involving LT-mediated epigenetic reprogramming, and might represent a novel mechanism by which this bacterium can evade the host innate immune response. This reprogramming would take place following spore germination and may compromise pulmonary host defense leading to bacterial proliferation and ultimate host death. Therefore, the use of pharmacological approaches to inhibit LT may represent a potential therapeutic strategy for the treatment of anthrax.

## Materials and Methods

### Animals and reagents

F-12K nutrient mixture, antibiotics, glutamine, Hanks' Balanced Salt Solution, Alexa Fluor® 488 F(ab′) fragment of goat anti-rabbit IgG (H+L) were from Invitrogen (Cergy-Pontoise, France). Fetal calf serum was from PAA (Etobicoke, USA). (TLR*grade*™) LPS from *E. coli* (Serotype 0111:B4) was from Alexis Biochemicals (San Diego, USA). Human β-actin antibody was from Sigma Aldrich (St. Louis, USA). SB203580 was from Tebu-bio (Le Perray, France). Antibodies against histone H2B (ab1790), histone H3 (ab1791), acetyl-histone H3 (H3AcK9, ab4441), methyl-histone H3 (H3me3K4, ab8580) and (H3me3K9, ab8898) and MSK-2 (ab42101) were from Abcam (Cambridge, USA). Antibodies against phospho-histone H3 (H3pS10, 05-817) and phospho-acetyl-histone H3 (H3pS10-AcK14, 07-353) were from Upstate Cell Signaling (New York, USA). Antibodies against RNA Polymerase II (RNAPII, sc-899), MEK-1 (sc-219), MEK-3 (sc-959) and the p65 subunit of NF-κB (sc-372) were from Santa-Cruz (Santa-Cruz, USA). Antibodies against MEK-2 (9125), p38-MAPK (9212), phospho-p38-MAPK (p38pT180-Y182, 9211), ERK (9102), phospho-ERK (ERKpT202-Y204, 9101), JNK (9252) and phospho-JNK (JNKpT183-Y185, 4668) and PD98059 were from Cell Signaling (Danvers, USA). Antibody against phospho-MSK-2 (Ser196), human and mouse recombinant TNFα were from R&D systems (Minneapolis, USA). Recombinant Lethal Factor (LF) and Protective Antigen (PA) from *B. anthracis* were from List Biological lab (Campbell, USA). Human bronchial epithelial cell line Beas-2B was from the American Type Cell Collection (Rockville, USA). Mouse alveolar epithelial cell line MLE-15 was a gift from Dr. J.A. Whitsett (Cincinnati Children's Hospital Medical Center, Cincinnati, USA). 7 weeks old C57/BL6 mice were supplied by the Centre d'Elevage R. Janvier (Le Genest Saint-Isle, France). Mice were cared for in accordance with Pasteur Institute guidelines, in compliance with the European Animal Welfare regulations.

### *B. anthracis* strains

The following isogenic non-capsulated *B. anthracis* strains were studied: the single mutant RP9 that produces active LF and inactive EF, and the double-mutant RPLC2 producing inactive LF and inactive EF. Both mutants produce functional PA [37].

### Mouse treatments

Mice were anesthetized by i.p. injection of a mixture of ketamine-xylazine as previously described [38] before intranasal instillation of 50 µl of bacilli (10^7^ cfu) or LPS (330 µg/kg). In certain experiments, anesthetized mice received intratracheal instillation of 10 µl of LT (550 µg/kg = 550 µg PA+550 µg LF, per Kg) 1 h before instillation of LPS or bacilli. Nascent bacilli were recovered from a 90 min growth in R medium as previously described [28].

### Incubation of cells with bacteria, toxins, and drugs

Epithelial cells isolated from lung homogenates as previously described [39],[40], Beas-2B and MLE-15 cell lines were pretreated with LT (PA+LF), PD98059, SB203580, or BAY for 1 h before incubation with LPS or TNFα at the concentrations and durations indicated in the figure legends. In other experiments, 1 h after addition of drugs or LT, Beas-2B cells were infected with bacilli for 3 h in the absence of antibiotics, washed and re-incubated for additional 24 h in a medium supplemented with antibiotics.

### Cytokine enzyme immunoassays and PMN counts

IL-8, KC, IL-6 and MIP-1 concentrations were measured in culture medium or in BALs using DuoSet ELISA kits from R&D systems (Minneapolis, USA). Total cell counts in BALs were determined with a Coulter Counter from Coulter Electronics (Margency, France), and differential cell counts were determined after cytospin centrifugation and staining with Diff-Quick products.

### Protein extraction and Western blot analyses

Proteins were extracted from Beas-2B cells using RIPA and then 20 µg of total proteins were subjected to SDS-PAGE. Proteins were transferred to membrane from Millipore (Billerica, USA), which were blocked and probed for 1 h with the indicated antibodies. After washing, the immunoreactive bands were visualized with a specific peroxidase-conjugated anti-immunoglobulin G (IgG) antibody and using an ECL Plus Western Blotting Detecting System (Amersham, Biosciences).

### Nuclear protein extraction and Electrophoretic Mobility Shift Assays

Nuclear proteins were extracted from Beas-2B cells, as previously described [41]. The NF-κB double-stranded oligonucleotides (Santa Cruz Biotechnology) 5′-AGT TGA GGG GAC TTTT CCC AGG C-3 γ-^32^P-labeled with T4 polynucleotide kinase (Biolabs) were incubated for 20 min at room temperature, with 5 µg of nuclear extract, 10 µl of binding buffer (40 mM HEPES (pH 7), 140 mM KCl, 4 mM DTT, 0.02% Nonidet P-40, 8% Ficoll, 200 µg/ml BSA, 1 µg of poly(dI:dC). Migration was performed on a 5% polyacrylamide gel in 0.5% Tris/borate/EDTA buffer at 150 V for 2 h. Gels were dried and exposed for 2 to 12 h.

### Immunofluorescence analysis

Beas-2B cells were fixed for 10 min with 3.7% (weight/volume) paraformaldehyde in PBS and then were permeabilized with 0.5% (volume/volume) Triton-X 100 in PBS. Fixed cells were treated 1 h with the indicated primary antibodies.

### Transfection studies

Beas-2B cells were cultured in 24 well plates, at 70% confluence and transfected with an IL-8 promoter construct [133]-Luc, an [Δ NF-κB] IL-8 promoter construct mutated on NF-κB site or a NF-κB construct-Luc. After indicated treatment, luciferase activity was measured using a luciferase reporter assay kit, with signal detection for 12 s by a luminometer (Berthold, Pforzheim, Germany),

### RNA extraction and real time Reverse-Transcription PCR analysis

Total RNA was extracted using an RNeasy kit from Qiagen (Courtaboeuf, France). DNase treatment was performed using 2 µg of extracted RNA, 1 µl of DNase I (Amersham Biosciences, Orsay, France), and 0.5 µl of RNasin (Promega, Madison, WI) in a total volume of 20 µl in the manufacturer's buffer. cDNA were obtained by incubating RNA with 1 mM dNTP (Eurobio, Les Ulis, France), 1.5 µl of hexamers as primers, 20 units of RNasin (Promega, Madison, WI), and 300 units of Moloney murine leukaemia virus reverse transcriptase RNase H minus (Promega, Madison, WI) in a total volume of 50 µl of the manufacturer's buffer for 1 h at 42°C and 10 min at 70°C. Real-time PCR was performed using the SYBR Green kit (Stratagene Brilliant II) and analyzed using the MxPro software (Stratagene, La Jolla, CA). The following primers were used. Proximal promoter of IL-8 (ppIL-8): sense 5′-AGTGTGATGACTCAGGTTTGCCCT-3; anti-sense 5′-AAGCTTGTGTGCTCTGCTGTCTCT-3′; proximal promoter of GAPDH (ppGAPDH): sense 5′ TCCCATCACCATCTTCCAGG-3′, antisens 5′-CATCGCCCCACTTGATTTTG-3′. Proximal promoter of KC (ppKC): sense 5′-GGAGCTCTGGAGTTTCGAGCATAA-3; anti-sense 5′-AGTCTGGAGTGCTGGAACTGGTTA-3′; KC-7 kb: sense 5′-TTCAAGCAGCCTCTCCCAGATCAA-3′, antisense 5′-CATTTGCCAGTCCTTTGTGGCTGA-3′. hCD44ex2: sense 5′-TGCCGCTTTGCAGGTGTATT-3′, antisense 5′-GGCAAGGTGCTATTGAAAGCCT-3′, mCD44ex2: sense 5′-TTTGAATGTAACCTGCCGCTACGC-3′, antisense 5′-AGGTACTGTTGAAAGCCTGGCAGA-3′. The PCR conditions were: 95°C 10 min, and 45 cycles at 94°C 15″ 60°C 30″ 72°C 20″, followed by the dissociation curve program for the analysis of the PCR products.

### Chromatin immunoprecipitation (ChIP)

Formaldehyde-fixed human Beas-2B and mouse MLE-15 cells were extracted to remove unfixed components. The average size of sonicated chromatin was approximately 500 base pairs, allowing analysis of the PCR products (150 base pairs; data not shown). Sonicated chromatin corresponding to 5.10^6^ cells was immunoprecipitated with 8 µg of irrelevant IgG or specific antibodies as indicated in corresponding figures. After extensive washing and reversed crosslinking, nucleic acids were used for quantitative real-time PCR, done with SYBR Green kits as detailed above.

### Control of cell viability

Cell viability was checked by the trypan blue dye exclusion test. Cell lysis was controlled measuring the release of lactate dehydrogenase (LDH) activity using a commercial kit from Boehringer (Mannheim, Germany).

### Statistical analysis

Data are expressed as the means±S.E.M. and statistical analyses were performed using an unpaired Student's t test or an ANOVA test. Statistical significances are indicated as * P<0.05; ** p<0.01; *** p<0.001.
